# Advances in Autophagy Regulatory Mechanisms

**DOI:** 10.3390/cells5020024

**Published:** 2016-05-13

**Authors:** Laura E. Gallagher, Leon E. Williamson, Edmond Y. W. Chan

**Affiliations:** Strathclyde Institute for Pharmacy and Biomedical Sciences, University of Strathclyde, Glasgow, Scotland G4 0RE, UK; Laura.Gallagher@Strath.ac.uk (L.E.G.); Leon.Williamson@Strath.ac.uk (L.E.W.)

**Keywords:** autophagy, amino acids, ULK, MTOR, autophagosome, isolation membrane, Beclin 1, VPS34, ATG14L

## Abstract

Autophagy plays a critical role in cell metabolism by degrading and recycling internal components when challenged with limited nutrients. This fundamental and conserved mechanism is based on a membrane trafficking pathway in which nascent autophagosomes engulf cytoplasmic cargo to form vesicles that transport their content to the lysosome for degradation. Based on this simple scheme, autophagy modulates cellular metabolism and cytoplasmic quality control to influence an unexpectedly wide range of normal mammalian physiology and pathophysiology. In this review, we summarise recent advancements in three broad areas of autophagy regulation. We discuss current models on how autophagosomes are initiated from endogenous membranes. We detail how the uncoordinated 51-like kinase (ULK) complex becomes activated downstream of mechanistic target of rapamycin complex 1 (MTORC1). Finally, we summarise the upstream signalling mechanisms that can sense amino acid availability leading to activation of MTORC1.

## 1. Introduction

All cells are challenged to adapt their metabolic pathways in response to nutritional levels and the resulting states of homeostasis go on to direct downstream decisions on growth, arrest or death. Macroautophagy, hereafter simply referred to as autophagy, is the intracellular recycling process that supports survival during times of energy stress and nutrient starvation. Under low nutrient conditions, cellular membranes elongate and sequester portions of the cytoplasm to capture a range of targets including proteins, organelles or foreign matter. This cargo capture event can be non-specific or directed by a family of adaptor proteins that recognise components labelled for degradation by ubiquitination. Engulfment of material then leads to formation of a nascent double-bilayer enclosed vesicle known as an autophagosome. Formation of the autophagosome represents early phases of autophagy in contrast to the late maturation stages that include the transport of autophagosomes and subsequent fusion with lysosomes or late endocytic compartments. Content mixing of autophagosomes and lysosomes marks transition to the degradative phase of autophagy in which metabolic building blocks are recycled back to the cytoplasm.

As a fundamental degradation and stress-response pathway, the function of autophagy is well conserved from plant and yeast, all the way through mammalian physiology. In humans, autophagy has become appreciated to play prominent roles in maintaining normal health at the cellular and organismal level from birth onwards, supporting essential homeostatic pathways that counteract slow deleterious events associated with aging [1,2]. The intracellular catabolism driven by autophagy influences an immense range of downstream functions in tissues, affecting outcomes in a number of medical fields including cancer, neurodegeneration and immunity, as reviewed elsewhere [3,4,5,6,7].

Research on autophagy can be historically traced back to the work of Christian de Duve during characterisation of the lysosome, leading to our current molecular era focusing on regulatory and membrane trafficking mechanisms [8]. While our understanding has steadily progressed, important questions still remain on how rates of autophagy are regulated in response to different extracellular cues and stress [9]. In many respects, it is helpful to be reductionist and consider just the rapid autophagy response following acute nutrient starvation, as this canonical form has been the most widely observed and is controlled by the core pathways. As the main nutrient-sensitive routes, starvation stress generally leads to activation of AMPK and inactivation of MTOR complex 1 (MTORC1) pathways [10]. AMPK and MTORC1 signals converge by phosphorylating and regulating the kinase, ULK1. ULK1 (or its close family member ULK2) represent the central components of the ULK1/2 autophagy regulatory complex that includes additional factors ATG13, ATG101 and FIP200 (RB1CC1). Once activated, the ULK1/2 complex drives initiation of autophagosome formation, leading to increased levels of autophagy. As such, the AMPK–MTORC1–ULK1/2 mechanism represents a critical upstream control point of the autophagy cascade and a key target for developing strategies to manipulate autophagy in biomedical contexts.

Discoveries in autophagy mechanisms continue to be uncovered even as we are now two decades post isolation of the initial yeast ATG genes. Undoubtedly, progress in mammalian autophagy has been essentially founded on studies of the homologous pathways in yeast and other model systems. From these concerted experiments, the field has defined how core features of the regulatory network are conserved while additional layers of complexity have joined during the evolution to higher organisms. Indeed, function of ULK1 in driving early stages of autophagy is directly parallel to roles of the homologous yeast ATG1 complex. Here, we aim to summarise three broad areas in mammalian autophagy that have undergone dramatic levels of revision in recent years. We discuss models for formation and expansion of autophagosome membranes. We next discuss how the ULK1/2 complex is activated to signal downstream autophagsome formation. Finally, we shift towards signalling mechanisms further upstream and discuss the emerging network of sensor pathways that link amino acid availability to the activation of MTORC1.

## 2. Formation of Autophagy Isolation Membranes

**Figure 1:**
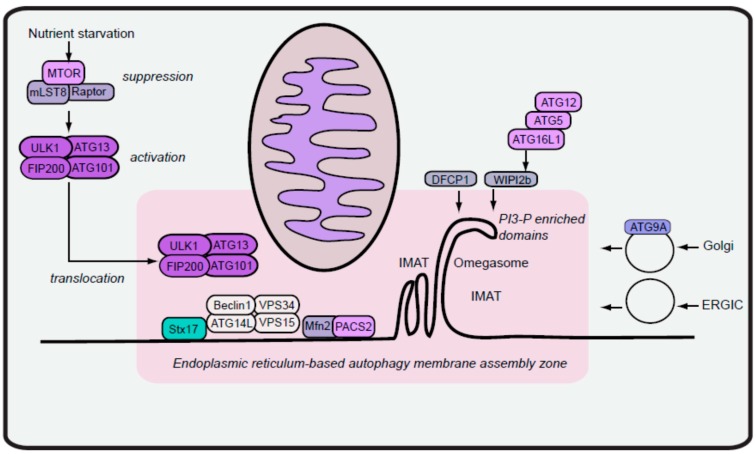
**Assembly of autophagy isolation membranes**. Starvation of nutrients leads to suppression of MTOR complex 1 resulting in downstream activation of the ULK1 autophagy initiation complex. The activated ULK1 complex translocates to an ER-based early autophagy membrane assembly zone associated with mitochondria contact sites, maintained in part via mitofusin 2 (Mfn2) and phosphofurin acidic cluster sorting protein-2 (PACS2). This assembly zone corresponds to early autophagy initiation puncta visualised by light microscopy. Activated ULK1 signals downstream by phosphorylating and activating the Beclin 1–VPS34–ATG14L–VPS15 complex, driving the generation of isolation membrane associated tubules (IMAT) and the omegasome. PI3P-enriched microdomains recruit markers like DFCP1 and machinery like WIPI2b along with further associated factors such as the ATG16L1–ATG5–ATG12 complex. After initial stages of membrane assembly, Golgi-derived vesicles containing ATG9A- and ERGIC-derived vesicles contribute further membranes and assembly machinery.

Once starvation signals are transmitted, the cell responds within minutes to increase formation of pre-autophagosomes, also known as isolation membranes (IM). These changes demonstrate a substantial rate of organelle biogenesis that is driven by a combination of membrane transport and remodelling. Since early morphological studies, the mechanism for autophagosome formation has been a fundamental question that still remains elusive in key details [11]. In yeast, autophagosomes are generated from a single perivacuolar membrane compartment termed the pre-autophagosomal structure (PAS) ([12]). In mammalian cells, however, the analogous IM assembly incorporates more complexity, with typically tens of autophagsosome initiation sites per cell upon activation. The key issues are: where are the initiation sites formed and from which source is the IM derived? These inter-related questions continue to be controversial, although the consensus is that autophagosome initiation sites are most often associated with microdomains of the endoplasmic reticulum (ER). The mechanisms of autophagosome formation have been reviewed in detail elsewhere [13,14,15]. Below, we summarise key features of ER-centralised autophagosome initiation and its stimulation by coordination of the ULK1/2 and Beclin 1/VPS34 complexes (summarised in Figure 1).

Earlier morphological and biochemical data from starved hepatocytes first indicated that ER was predominantly associated with autophagosomes [11]. Since these observations, evidence with fluorescent-tagged autophagy molecular components further supports a close link between IM formation and the ER. One significant advance was the demonstration of autophagosome assembly on regions of membrane derived from the ER marked by the protein DFCP1 (double FYVE domain-containing protein 1) [16]. Via its phosphatidylinositol-3 phosphate (PI3P) binding FYVE domains, DFCP1 was observed to translocate onto distinct PI3P-enriched regions of the ER following amino acid starvation and autophagy induction. Because these PI3P-containing DFCP1-labelled structures had a cup shape (like the uppercase letter-Omega), they were named omegasome membranes. While the functional role of DFCP1 for autophagosome formation remains enigmatic [16], DFCP1 remains a robust marker of IM initiation sites in a range of cell models.

### 2.1. Early Isolation Membranes from ER-Mitochondria Contacts

Ultrastructural studies have helped characterise the intimate spatial relationship between IM initiation sites and the ER. Conventional transmission electron microscopy first suggested this close association [17,18], which has been resolved to finer detail using 3D electron tomography. For example, in amino acid-starved normal rat kidney (NRK) cells, multiple direct interconnections could be observed between the ER and forming autophagosome, in particular near the growing edge of the IM [19]. In addition, tomography has been able to reveal intimate structural features, such as the close association between the IM/ER interface and lipid droplets, consistent with proposed roles for lipolysis-derived neutral lipids to support autophagosome membrane expansion [20,21]. Tomography carried out using NIH3T3 cells revealed how cup-shaped bilayer regions of the ER can partially support both sides of the forming IM, leading to the conception of the ER cradle model for autophagosome formation [22]. Other experiments combining electron microscopy and tomography have characterised the presence of DFCP1-positive 30 nm tubular membrane structures associated with the ER that join to edges of the IM [23]. These IM-associated tubules (IMATs) were widely observed, for example, in HeLa, retinal epithelial, hepatoma and NRK cell lines. Moreover, IMAT could be detected in ATG3, ATG5, ATG7 or ATG16L1 −/− MEFs, which are deficient in different components of the autophagy ATG8/LC3 conjugation pathway. In contrast, IMAT formation was blocked in MEFs lacking the FIP200 component of the ULK complex. These data support a model in which early IM formation, driven by tubulated ER projections, precedes involvement of membrane fusion events driven by activated ATG8 proteins. The common aspect was that membrane assembly was occurring on or in direct proximity to the cradle-shaped omegasome sites associated with DFCP1, and importantly, ULK/FIP200 function was essential for this step.

What are the upstream pathways signalling IM formation? Organisation of DFCP1-omegasomes are driven by increases in local concentrations of PI3P, formed by the VPS34 Class III PI3-kinase. VPS34 is part of the Beclin 1 autophagy signalling complex, which includes subunits ATG14L (also known as Barkor) and p150/VPS15 [24]. The multi-functionality of the Beclin 1 complex needs to be noted. While the core complex containing ATG14L drives autophagy membrane initiation, additional related Beclin 1 complexes are formed through modulatory subunits UVRAG, Rubicon and Ambra1 to regulate distinct trafficking stages of the formed autophagosome or endosome to the lysosome [25,26]. As expected, inhibition of ATG14L or its correct translocation to the ER upon starvation prevents formation of omegasomes [27]. Correct targeting of ATG14L is also key for anchoring Beclin 1/VPS34 complexes at IM assembly sites on ER microdomains. A conserved region within ATG14L, termed BATS (Barkor/ATG14L autophagosome targeting sequence), has been identified, that preferentially binds PI3-P and curved membranes [28]. As such, ATG14L may also have structural roles in shaping or stabilising the forming IM. Interestingly, other cell biology and biochemical approaches suggested that mitochondria were also critical in providing lipids for autophagosomes [29]. Further analysis of ATG14L during membrane assembly was able to provide some clarification in the model by highlighting the involvement of ER-mitochondria contact sites during autophagy initiation [30].

The ER is a vast membrane network, serving as the primary location for synthesis of proteins to be targeted towards the Golgi and further downstream secretion or lysosome maintenance. The ER shares connectivity with the nuclear envelope and most other major classes of cellular membranes, including mitochondria. ER-mitochondria contact sites have recently become better characterised as membrane microdomains with specific regulatory factors, enzymatic activities and functions for lipid and calcium homeostasis [31]. Components of ER-mitochondria contact sites are particularly enriched in mitochondrial-associated ER membranes (MAM), a biochemical fraction associated with markers such as the enzyme fatty acid CoA ligase 4. With this biochemical approach, it was first noted that ATG14L was not present in MAM isolated from cells maintained under full nutrients. Upon starvation, ATG14L, along with DFCP1 and other subunits of the Beclin 1/VPS34 complex, became enriched in the MAM, indicating a dramatic nutrient-dependent recruitment of autophagy factors. Live-cell imaging, indeed, could show ATG5, a marker for forming IM [32], associating with ER and mitochondrial markers. Quantification revealed that ATG5-labelled IM were almost entirely associated with ER markers, while association with the mitochondrial marker was dynamic and transient. These observations are consistent with a refined model in which the ER forms the predominant stable scaffold for forming IM, with more rapid membrane contributions from mitochondria contacting IM assembly sites. Knockdown of genes critical for the organisation of ER-mitochondria contact points (such as phosphofurin acidic cluster sorting protein-2 or mitofusin 2) blocked proper translocation of ATG14L and formation of functional autophagosomes. Interestingly, knockdown of the ER-associated SNARE protein, syntaxin 17, also disrupted proper localisation of ATG14L at ER–mitochondria contacts and an accumulation of arrested autophagy membranes. Thus, SNARE syntaxin 17 has important organisational roles for the ATG14L complex during the early stages, before acting at later during autophagosome maturation [33].

Compilation of all data leads to a consensus mechanism. Assembly of ATG14L-anchored Beclin 1/VPS34 complexes at the ER–mitochondria interface generates concentrated pools of PI3-P that then drive membrane tubulation to form and expand the IM. Lipid-dependent IM formation depends on PI3-P interacting effectors such as members of the beta-propellers that bind phosphoinositides (PROPPIN)/WIPI protein family (summarised elsewhere [34]). The WIPI2b member, specifically, has been shown to have a downstream effector mechanism by binding ATG16L1, thus recruiting the ATG5–ATG12–ATG16L1 oligomeric complex to the initiation site [35]. The ATG5–ATG12 moiety of the complex in turn promotes lipidation of ATG8/LC3 by directly binding and stimulating activity of ATG3 [36,37]. As such, a step-wise mechanism is formed linking Beclin 1/VPS34 recruitment, PI3-P generation and ATG8 activation.

### 2.2. Maturation of Isolation Membranes via Vesicles from ER Exit Sites

Overall, the model with ATG14L–Beclin 1–VPS34 complexes on ER–mitochondria contact sites may reflect the central pathway for IM initiation. Multiple pathways likely feed into this basic scheme during the elongation phase forming the complete autophagosome. Much evidence supports the contribution of a wide range of cellular membranes (besides the ER and mitochondria) towards autophagosomes, including endosomes, the Golgi and the plasma membrane [38,39,40,41]. The long existing controversy on this topic seems to indicate that autophagosome formation involves the core pathway, with ER-based initiation, coupled with additional non-mutually exclusive membrane interactions that drive membrane growth to form the complete autophagosome. Other work has further suggested that ER exit sites (ERES) may play a critical role for autophagy, particularly at the elongation stage ([42], and reviewed in [43]). ERES are specialised platforms on the ER where proteins are sorted into COP II coatomer vesicles for downstream trafficking to the Golgi [44]. In yeast, mutation of SEC12 (GEF for the Sar1 GTPase) disrupts ERES and prevents proper localisation of ATG14 and downstream assembly steps [45].

Studies of mammalian autophagy have highlighted particularly important functions for the ER–Golgi intermediate compartment (ERGIC) [46], which is the membrane structure receiving vesicles from the ERES for further trafficking towards the *cis*-Golgi. Cell-free biochemistry approaches that reconstituted the ATG8/LC3 lipidation step indicated that membranes from the ERGIC fraction were enriched in LC3 lipidation activity, representing the presence of ATG5–ATG12–ATG16L and ATG3. In contrast, MAM fractions did not contain any detectable LC3 lipidation activity. These data suggest that IM may initially form from ER–mitochondria exit sites, while ATG8 lipidation activity joins later via transport vesicles from the ERGIC. Generation of COP II vesicles carrying ATG8/LC3 lipidation activity is dependent on PI3-P formation [47]. As such, the ATG14L–Beclin 1–VPS34 complex has been proposed to remain associated to IM initiation sites that then later mature into membranes associated with ERES and ERGIC. An alternative is that ATG14L–Beclin 1–VPS34 joins into COP II vesicles *en route* to the ERGIC, but current evidence cannot discriminate between these two possibilities.

The model above features IM from ER–mitochondria contact sites gaining further membranes and autophagy machinery via ERGIC-derived vesicles. While pared down schemes can be useful, it is widely acknowledged that multiple pathways are needed for a holistic view of autophagosome formation. Morphological analysis of membranes in starved NRK cells shed further light on the relative balance of pathways involved, even within the same cell type and stimulus [48]. Quantification of immuno-electron microscopy has aimed to measure sites of LC3-labelled newly formed autophagosomes. In agreement with the emerging model, early autophagosomes were observed most frequently with ER (59%). Autophagosomes were also observed in proximity with other membranes, but with lower frequency (close to: mitochondria (22%), recycling or late endosomes (16%) and Golgi (14%)). These data are consistent with live-cell imaging indicating that IM form with most stable support from the ER with additional transient contact of mitochondria [30]. The fewer instances of Golgi overlap may reflect lower relative amounts or shorter half-lives of these contacts, while the co-localisation with late endosomes may represent the maturation stage. Higher resolution tomography could reveal further clues, specifically on early IM formation. These data similarly supported the ER as the foundation membrane for autophagy initiation. In addition, tomography captured further evidence for IM closely tethered to a range of cellular membranes, in some cases with one IM simultaneously contacting multiple types of membranes. More strikingly, 100% of IM observed were in close proximity to the ER. IM were associated, but less frequently, with mitochondria (29%) and endosomes/lysosomes (17%), while IM were rarely captured next to ERES (0.5%) or Golgi (0.5%), which could represent either fast membrane contacts or relatively lower contributions.

### 2.3. Outlook on Autophagy Membrane Initiation

To conclude, the data together support a model in which IM are formed from an initial ER-based membrane site (Figure 1). Following, multiple types of membranes dynamically interact with the ER to further donate membranes for IM initiation and subsequent elongation, although mitochondria and endosomes seem to be the major contributors. Vesicles from the ERGIC donate membranes and further factors such as ATG8 lipidation machinery to the forming autophagosome. Rapidly trafficking vesicles containing ATG9A (also known as mATG9) also contribute essential membranes to the IM [49,50]. The ATG9A pathway provides a mechanism to incorporate traffic from endosomes, Golgi and the plasma membrane biogenesis [40,51]. A recurring theme is that factors can play key roles at multiple steps. The ER SNARE syntaxin 17, appears to coordinate events at IM formation, autophagosome completion and degradative maturation. Vacuolar membrane protein 1 (VMP1) is another ER protein that may function at both early and later stages of autophagy [49,52]. ATG14L, which has been highlighted here to assemble Beclin 1/VPS34 complexes to initiate the IM, plays additional roles during maturation via syntaxin 17 [53]. ATG5 is a protein that assembles early at IM formation sites [32], yet appears to be essential for later elongation stages [49], possibly by promoting ATG8 lipidation activity via interactions with ATG3 [37]. The ATG3 pathway can further incorporate membrane-sensing mechanisms, directing ATG8-lipidation activity towards highly curved bilayers such as on the IM edges [54]. In this regard, a number of pathways are likely necessary to help properly shape the curved membranes encountered during autophagy. The BATS domain in ATG14L can provide structural support at highly curved bilayers or tubules [28]. Localised punctate assemblies of polymerised actin may also help provide scaffolding to shape IM initiating from the ER [55]. While IM formation is ER-based, other data highlight that autophagy can be turned around on itself during ER-phagy—to capture and degrade portions of the ER via receptor-mediated mechanisms [56,57]. Importantly, all models for autophagosome formation feature a central role for PI3-P. Reflecting this, there has been long-running focus on targeting VPS34 as a strategy to inhibit autophagy, even though this approach would be expected to affect a wide range of other vesicular pathways. Compounds such as wortmannin, LY294002 and 3-methyladenine have been generally effective in blocking autophagy, but there was a critical lack of specificity since all these agents can target class I PI3 kinases in addition to VPS34. On this front, recent work has led to the development of several specific and potent VPS34 inhibitors such as VPS34-IN1 (25nM IC_50_), PIK-III (18 nM IC_50_) and SAR405 (IC_50_ 1.2 nM) [58,59], which should be more precise tools to target VPS34-dependent autophagy (as reviewed further in [60]). The elucidation of key structural features within the VPS34 complex will further facilitate refinement of compounds and their mechanisms [61].

## 3. Regulation and Role of the ULK1 Complex during Autophagy Initiation

### 3.1. Phosphorylation of ULK1

In considering upstream pathways, several core mechanisms link ULK1 to the regulation of VSP34 during autophagy. Since activity of the ULK1/2 complex is coordinated by AMPK and MTORC1, signalling steps directly connect nutrient-dependent cues to autophagy initiation at the ER. The related family members, ULK1 and ULK2, show strong sequence similarity and appear to be functionally redundant *in vivo* [62,63], although regulatory events still remain better characterised for ULK1. In the current model, amino acid starvation leads to inactivation of MTORC1 and lower levels of phosphorylation on ULK1. MTORC1-mediated phosphorylation of ULK1 produces inhibition, in part by inducing conformational changes that prevent ULK1–AMPK interactions [64]. AMPK-dependent phosphorylation of ULK1 has the overall effect of promoting function in the ULK1 complex [64,65]. While this mechanism provides a framework for understanding ULK1 and its regulation by nutrients, the model does not fully incorporate the full range of complexity that feeds into the pathway. In terms of post-translational modifications, over 70 phosphorylation sites are currently listed for mammalian ULK1 in databases (e.g., phosphosite.org). Phosphorylation events have been detected along all three major domains of the protein (N-terminal kinase, internal spacer region, and the C-terminal early autophagy targeting (EAT) domain), which represent the action of a number of kinases in addition to auto-phosphorylation. With this vast set of modifications, understanding of the functional roles of specific sites is still lacking and it is unclear how all the modifications are coordinated, as summarised elsewhere [66]. In addition, ULK1 can also receive ubiquitination and acetylation modifications during autophagy and these need to be involved in the overall model [67,68,69,70,71].

### 3.2. Regulation of ULK1 by MTORC1

At present, some clarity is gained by focusing just on the ULK1 sites regulated by MTORC1 and AMPK, which have been the best characterised. MTORC1 phosphorylates ULK1 on serine 757 (using mouse ULK1 amino acid annotation (PVVFTVG**S**PP)) and levels of this modification closely correlate with MTORC1 activation under amino acid-replete conditions [64,72]. ULK1-S637 (mouse annotation) is the other known MTORC1-regulated site [72,73]. Currently, modifications on ULK1-S757 have been the most widely reported, both in cell models and *in vivo*, with decreased P-S757 generally correlating with autophagy activation [74,75,76,77,78,79,80]. Conversely, ULK1-S757 is phosphorylated in response to type-I interferon signalling [81]. Functionally, mutation of S757 alters the kinetics of the autophagy response [72] and also decreases ULK1–AMPK binding [64], suggesting a model in which MTORC1 regulates the ability of AMPK to bind and activate ULK1.

An interesting development has been the identification of protein phosphatase 2A (PP2A) as a regulator for ULK1, specifically on the S637 site [73]. Wong *et al.* first noted that nutrient starvation triggered a more rapid dephosphorylation of the MTORC1 sites on ULK1 (and more rapid autophagy) as compared to MTORC1 inhibitory drugs. Rapamycin or Torin1 treatments, which are widely used tools, did indeed induce autophagy and changes on the ULK1 sites, but not as robustly as acute nutrient starvation. These observations thus suggested a starvation-induced phosphatase that dephosphorylated the MTORC1 sites to trigger autophagy, which was subsequently identified to be PP2A.

The inhibitory effects of okadaic acid on autophagy have indeed been observed earlier in studies of hepatocytes and neurons [82,83]. Other work from yeast, *Drosophila* and *C. elegans* systems lend further support for a conserved pathway linking PP2A to ATG1-dependent autophagy [84,85,86,87]. Wong *et al.* build upon these earlier studies to define the nutrient-dependent mechanism directing PP2A activity to ULK1 dephosphorylation. Since specificity of PP2A catalytic activity is controlled by its associated regulatory B-subunit, and at least 15 B isoforms are present in the human genome (see [88] for review), it was an important further advance that the authors were able to identify B55-alpha as the key regulatory subunit directing activity onto ULK1. The starvation-dependent mechanism for PP2A regulation involved a further factor. Activity and stability of PP2A are regulated in part via interaction with the alpha 4 protein (Tap42 in yeast) that keeps the catalytic subunit in an inactive state and prevents ubiquitination [89]. Nutrient starvation disrupted interactions between alpha 4 and PP2A catalytic subunits and this effect could not be mimicked by Torin1 treatment. Thus, only a starvation signal is able to promote PP2A activity, ULK1 dephosphorylation and the maximal, rapid autophagy response. Consistent with this model, mutation of Tap42 is able to induce ATG1-dependent autophagy in yeast [87]. The work on PP2A has also been valuable for shedding light on the relative roles of the ULK1-S637 and -S757 sites. Okadaic acid produced a clear effect, blocking the formation of autophagosomes. However, okadaic acid primarily inhibited dephosphorylation on the S637 site, while changes on S757 were not affected. These data suggest that dephosphorylation of S637 may be the key regulatory signal during formation of autophagosomes. Since the other MTORC1-sensitive site, S757, has potential to modulate interactions with AMPK [64] and autophagy [72], several non-mutually exclusive mechanisms may integrate multiple signalling pathways. Combined phospho-mimetic or phosphorylation-incompetent mutations at these 2 MTORC1 sites might create constitutively inactive- or active-ULK1, respectively, although this needs to be shown experimentally and may be an oversimplification in light of the other modifications on ULK1.

### 3.3. Regulation of ULK1 by AMPK

Binding of AMPK to ULK1 has been widely observed, both from focused and larger proteomic approaches [64,72,90,91,92,93]. The AMPK–ULK1 interaction leads to a more complex set of modifications, with phosphorylation of at least seven serine/threonine residues in ULK1 [64,65,72]. It remains unclear how all AMPK signals integrate together to regulate ULK1, as all combinations from multi-site modification have not yet been explored. Simultaneous mutation of four AMPK sites within the ULK1 internal spacer region (mouse S467, S555, T574 and S637) impaired cellular responses including mitophagy and survival following prolonged nutrient stress [65]. Phosphorylation on S555 site controls direct binding to 14-3-3 proteins and ULK1 function, for example, during regulation of ATG9A trafficking [65,92,93]. In a separate study, dual mutation of sites ULK1-S317 and -S777 impaired the autophagy and cell survival response following prolonged glucose starvation [64]. Of these AMPK sites, modification of ULK1-S555 has so far been most widely observed, correlating with AMPK activation following glucose starvation, pharmacologic agents, or knockdown of the MAGE-A3/6-TIM28 ubiquitin ligases that down-regulate AMPK [65,76,92,94,95].

Consistent with earlier work on ULK1-S555, more recent results further show how this site is phosphorylated by activated AMPK following hypoxia stress to direct mitophagy [96]. Phosphorylation of S555 played a role in directing translocation of ULK1 onto mitochondria following hypoxia, and mutation of S555 blocked this regulation. Moreover, ectopic expression of an ULK1-S555D active variant could drive constitutive mitophagy, indicating a mode of directing ULK1 localisation by just a single AMPK site. Once on damaged mitochondria, the active ULK1 complex drives mitophagy by phosphorylating the mitochondrial resident protein FUNDC1 on serine 17 [97]. Phosphorylation of FUNDC1 promotes its interaction with LC3, providing one mechanism underlying increased ULK1-dependent mitophagy. Another mitophagy mechanism involves ULK1-mediated phosphorylation of ATG13 on serine 318 [98]. This phosphorylation signals for ATG13 to dissociate from the ULK1 complex and translocate onto damaged depolarised mitochondria to promote parkin-dependent mitophagy. In this context, ATG13 plays a more direct role during the mitophagy response, as compared to ULK1 which does not translocate to mitochondria following protonophore-mediated mitochondrial damage. Interestingly, ATG13-specific roles have also been suggested in TNF alpha-induced caspase-dependent cell death from studies of an ATG13 knockout model [99], although this could represent another pathway unrelated to mitochondria. Taken together, several distinct ULK1-mediated mitophagy mechanisms thus appear to control the response, depending on the severity of damage.

So far, the data on the S555 and S637 sites have illustrated the potential of phosphorylation to control sub-cellular localisation of ULK1. In this regard, regulation of ULK1 localisation has been a longstanding question in the autophagy field. Following acute nutrient starvation, the ULK1 complex rapidly translocates to punctate ER-associated autophagosome/IM assembly sites and this event is considered an early checkpoint during the initiation cascade [32,52]. Translocation has been widely observed for ULK1 and other components of the complex following amino acid starvation [52,100,101,102,103,104,105,106]. In mammalian cells, ULK1 is bound to its co-factors ATG13, FIP200 and ATG101, forming oligomers with a molecular weight near three megaDaltons [102]. Furthermore, this higher-order assembly was not altered following starvation. As such, the ULK1 complex was not remodelled or disassembled during autophagy and so post-translational signalling thus seemed critical.

How could modifications on ULK1 drive translocation? Phosphorylation of S555 and S757 can modulate protein interactions [64,65,92]. Analysis of kinase-inactive mutants also suggested that ULK1 is a protein that can adopt different conformations [103]. Speculatively, phosphorylation of ULK1 on S555 may cause a conformation that interacts with factors on damaged mitochondria. Dephosphorylation of S637 might signal a distinct conformation that favours association with starvation-induced IM sites on the ER. It remains unclear what may constitute ULK1 interaction targets during the distinct mitophagy and autophagy initiation recruitment steps. For nutrient-dependent autophagy, one candidate is the protein VMP1, which is localised at punctate sites on the ER that precede recruitment of the ULK1 complex [32]. Interaction with protein factors may bring specificity that combines with the intrinsic membrane-binding activities of the ULK1 complex. The C-terminal EAT sub-domain of ULK1 is both necessary and sufficient to bind membranes and localise to autophagosomes [101,103]. Lipid-binding residues in mammalian ATG13 have been identified [105] and FIP200 can provide further recruitment activity by directly binding ATG16L [107,108]. Functional evidence supports all these mechanisms, but relative contributions of each are unclear and may be context-dependent. Interestingly, both ATG13 and FIP200 are phosphorylated via ULK1- and MTORC1-dependent mechanisms [98,100,102,109,110,111]. These signals might promote recruitment of ATG13 and FIP200, but the functions of specific phosphorylation sites in these proteins are still not defined.

### 3.4. Outlook on ULK1 Regulation

While translocation of the ULK1 complex to membrane sites has been widely observed, it remains unclear how catalytic function of the complex is activated. Activation of ATG1 kinase activity following nitrogen starvation of yeast could be clearly detected [109]. More recently, activation of ULK1 in cells following starvation could be detected, for example, by probing phosphorylation of the S318 site of ATG13 [41,111]. Specific modifications (e.g., phosphorylation) that are sufficient to drive ULK1 catalytic activity have not yet been identified. However, additional associated proteins have emerged with potential to regulate ULK1 kinase activity. In one model, the protein huntingtin has been shown to bind ULK1 and this interaction was mutually exclusive with MTORC1–ULK1 binding [112]. Thus, it was proposed that huntingtin would compete and prevent MTORC1-mediated inhibition of ULK1, thereby leading to ULK1 activation. An additional model has been proposed highlighting how ULK1 activity may require other regulatory interacting proteins, such as the GABARAP member of the mammalian ATG8 family [41]. The ULK1–GABARAP mechanism utilises a dynamic interplay of interactions that involves formation of an inactive complex containing GABARAP and GM130 localised on the Golgi. An additional protein, WAC, is then able to shift GABARAP to a distinct active pool that binds and activates ULK1 kinase function to promote autophagy. Several features of this model are unexpected, for example, that binding of ULK1 was specific for the non-lipidated form of GABARAP and did not involve other ATG8 family members. Also, ULK1–GABARAP binding was not starvation-dependent leading to a model in which translocation of the ULK1–GABARAP complex to the IM site is the critical event to drive autophagosome formation forward. Thus, all the evidence indicates that ULK1 function is controlled via the concerted action of post-translational modification, interacting proteins and sub-cellular translocation.

Future progress in ULK1 mechanisms may be further driven by structural elucidation of the components following advances for yeast ATG1. The regulatory mechanisms between yeast and mammalian ATG1/ULK1 are not identical but key features are conserved. Consistent with mammalian ULK1, the budding yeast *Kluyveromyces* EAT domain is able to bind and remodel liposomes in membrane tethering assays [113]. Also, the EAT domain of both yeast and mammalian ATG1/ULK1 binds its corresponding ATG13 co-factor [103,110,114]. The crystal structure of this interaction has been solved using *Kluyveromyces* ATG1 EAT in complex with an ATG13 sub-domain and these results highlight a six alpha-helix fold within the EAT, modelled to resemble tandem MIT (microtubule interacting and transport) domains [115]. A solution structure has been solved for the pentameric complex containing the ATG1 EAT and portions of ATG13, ATG17, ATG31 and ATG29 from budding yeast *Lachancea thermotolerans* [116]. All these data have helped form a model in which the pentameric complex oligomerises as a scaffold at the yeast PAS. Single-particle electron microscopy has been an independent approach to study purified pentameric complexes from budding yeast *S. cerevisiae* [117]. This complementary approach has been able to visualise the S-shaped ATG17–ATG31–ATG29 dimeric complex and further refine positioning of interactions with the ATG1–ATG13 subcomplex.

It remains unclear the extent that mammalian ULK1 structures might resemble the pentameric networks proposed for yeast ATG1. This issue is beginning to be resolved through studies of the mammalian ATG13–ATG101 HORMA (Hop1/Rev7/Mad2) domains in complex. Comparison of the human and fission yeast *S. pombe* crystal structures has revealed that overall architecture and interaction interfaces are conserved [118]. The *S. pombe* structure was used as a basis to successfully design mutants in human ATG101 that then disrupted ATG13-binding and autophagy function [106]. It has been speculated that the ATG13–ATG101 HORMA interaction found in *S. pombe* may represent the ancestral form of this functional module which has been conserved all the way to mammals [118]. The accumulating evidence suggests that, through evolution, budding yeast lost function of ATG101, while gaining novel regulatory factors such as ATG29 and ATG31, in addition to other mechanisms not found in mammals [106,119]. As such, mammalian ULK1 structure may be more similar to *S. pombe* ATG1. Mammalian ULK1 and ULK2 are between 1036–1051 residues in length, which has so far brought challenges in structural studies of full-length proteins. However, the crystal structure of the ULK1 kinase domain in complex with an ATP-site inhibitor has been solved [120]. In this context, the kinase structure was useful for rationale design of more potent inhibitory compounds and this approach should serve as a basis for future refinement of compound specificity.

### 3.5. Downstream Targets of ULK1

**Figure 2:**
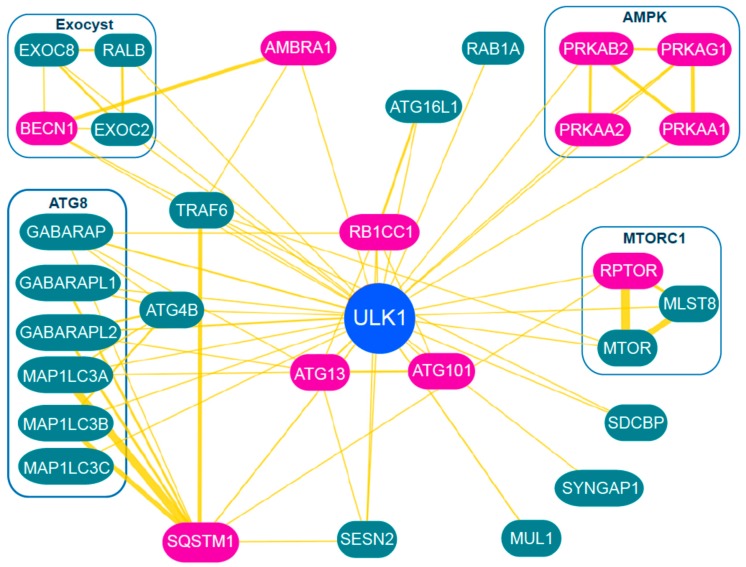
**Summary of the ULK1 interactome**. A subset of ULK1 protein interactions collated by the BioGRID server was selected for representation. Proteins characterised to also serve as ULK1 substrates are highlighted pink. Thickness of connectors corresponds to numbers of experimental entries supporting interaction. Note: this interaction database does not capture all known ULK1-binding partners including, for example, ATG9, ATG14L1 or FUNDC1.

Once the ULK1 complex translocates to membrane-associated assembly sites, downstream substrates of ULK1 are phosphorylated to signal autophagy activation. Kinase-inactive ULK1 does not promote, but rather inhibits autophagy, indicating that downstream phosphorylation is critical [101,103]. Several ULK1 kinase inhibitors (for example MRT68921 and SBI-0206965) have recently been developed that block autophagy, bringing the field closer towards targeting this pathway as a therapeutic [74,111,120]. Another major question is: how does ULK1 signal autophagy downstream? The full range of ULK1-directed pathways will be driven by downstream effector molecules and interaction partners. Protein interaction databases [121] collating low- and high-throughput data currently list over 40 binding partners for ULK1 (summarised in Figure 2), which include GABARAP, p62/Sequestosome1 (SQSTM1, and the MTORC1 and AMPK complexes. Such dynamically updated databases will be useful to organise and visualise the networks of primary strong core interactions and more transient regulatory binding partners. A large number of substrates have already been reported for ULK1 (summarised in [66]). Overall, the large set of proposed ULK1 substrates overlaps substantially with the ULK1 interactome. These include factors from (or interacting with) autophagy-signalling networks such as p62, AMBRA1, AMPK and RAPTOR [122,123,124,125]. ULK1 has been reported to phosphorylate other autophagy regulatory proteins such as ATG9, ZIP kinase and folliculin [126,127,128]. Substrates for *C elegans* unc-51 (homologue of ATG1/ULK1) have been suggested, such as unc-14, unc-76 and VAB-8L, which function during endocytic trafficking [129,130]. ULK1 also phosphorylates STING (stimulator of interferon genes) to limit its activity as part of a negative feedback mechanism [94]. As mentioned above, ULK1 phosphorylates ATG13 and FUNDC1 during the mitophagy response [97,98].

### 3.6. ULK1 Phosphorylation of the Beclin 1–ATG14L–VPS34 Complex

Which ULK1 substrate promotes IM formation? On this point, a major step was the finding that ULK1 phosphorylates and activates Beclin 1 during autophagy induction [131]. The modification was identified on serine 14 (S15 in humans) which was conserved in other Beclin 1 homologues, for example, from *Drosophila* and *C elegans*. Phosphorylation of S14 correlated with upstream signalling activity and was increased following starvation or MTORC1 inhibition. S14 phosphorylation was important to promote full activation of associated VPS34 to produce PI3P. Furthermore, mutation of S14 to alanine blocked Beclin 1 function during autophagy, while conversely, a phospho-mimetic mutant at position 14 was sufficient to drive autophagy. ATG14L played a critical role in this mechanism by binding ULK1, promoting ULK1–Beclin 1 interaction and the subsequent phosphorylation event. Moreover, mutation of the BATS membrane-targeting domain blocked the ability of ATG14L to regulate the ULK1–Beclin 1 mechanism, which suggested that the ULK1 and ATG14L-Beclin 1 complexes needed to be interacting together at the autophagosome assembly site in order for the phosphorylation to occur. Overall, these findings defined a clear mechanism linking nutrient-dependent signalling to ULK1 and downstream activation of Beclin 1–ATG14L–VPS34.

A following study further highlighted additional mechanisms that regulate the ULK–VPS34 pathway. As the starting point, these experiments focused on ATG13 and its role in stabilising interactions between ULK1 and Beclin 1 complexes [132]. Mapping approaches indicated direct binding between the N-terminal HORMA domain of ATG13 and an internal region of ATG14L, consistent with the ATG13–ATG14 interaction detected in yeast [133]. The bridging function of ATG13 promoted ULK1-dependent phosphorylation on ATG14L, as seen by electrophoretic mobility shifting. A number of phosphorylated sites could be detected on ATG14L by mass spectrometry, but further work with mutagenesis and phospho-specific antibodies so far only focused on the serine 29 site, which was responsive to starvation or MTORC1 inhibition. Phosphorylation on ATG14L-S29 could be detected in mouse tissues, which was also responsive to dietary modulations, validating this pathway *in vivo*.

Functionally, phosphorylation on S29 was required for full starvation-dependent activation of VPS34 and autophagy. Consistent with a key role, ATG14L with an activating mutation at position 29 was also able strongly stimulate activity of associated VPS34. Modification of S29 might possibly induce a conformational change in ATG14L that then activates VPS34. However, the ATG14L-S29D-activating mutation only partially promoted autophagy indicating that other cooperating mechanisms are required for a full response. Considering the findings so far, multiple pathways seem to integrate towards VPS34 regulation. Further work indicated that Beclin 1 phosphorylation functioned independently from that of ATG14L and so both these ULK1-driven phosphorylation events may synergise for combined spatial and catalytic regulation of VPS34. Roles for the other sites phosphorylated in ATG14L are still unclear. ATG14L is also phosphorylated by MTORC1, leading to suppression of associated VPS34 and autophagy [134].

Overall, details are still unclear on how the multiple signalling mechanisms coordinate in order to control autophagy. However, the predominant model shows MTORC1 and AMPK converging toward the regulation of ULK1 leading to subcellular translocation and downstream activation of the Beclin 1–ATG14L–VPS34 complex. Additionally, we need to keep in mind other key data that demonstrate distinct VPS34 functional complexes [135]. VPS34 complexes containing Beclin 1, along with either ATG14L or UVRAG, directed autophagy. On the other hand, VPS34 alone or in complex with just Beclin 1 regulate distinct pathways in vesicular trafficking or cellular stress responses. In this system, AMPK specifically regulates the non-autophagy pathway by phosphorylating VPS34 (on T163/S165). AMPK activated via glucose starvation also regulates the pro-autophagy complexes by phosphorylating Beclin 1 (on a distinct set of sites: S91 and S94). Beclin 1-S91 and S94 may also be sensitive to amino acid starvation pathways [136]. As such, key questions still remain as to how the multiple pathways feed into the Beclin 1–VPS34 complex together. In fact, the wider scope of evidence indicates that Beclin 1 forms a pleiotropic-signalling hub that integrates phosphorylation events from AMPK and ULK1, in addition to AKT, DAPK, ROCK1, MAPKAPK2/3 and the EGF receptor [137,138,139,140,141].

### 3.7. Outlook on Signalling Downstream of ULK1

From all available autophagy data, key features of the MTORC1, AMPK, ULK1 and VPS34 nutrient-dependent mechanism have now become better defined leading all the way to formation of the initiation membrane. What is now needed in this area? The field already has some pharmacological approaches for inhibiting ULK1/2 [74,111,120] and these tools could be further refined. To interpret effects of inhibitors *in vivo*, we need to understand the full range of pathways regulated by ULK1. Is ULK1 differentially regulated to control non-specific starvation-induced autophagy *vs.* specific pathways like mitophagy and xenophagy? How does ULK1 coordinate autophagy with roles in other pathways such as growth regulation and vesicular trafficking [130,142,143]? The range of ULK1 functions will be related to its interactome and set of substrates, but the full scope of these pathways is unclear. Along this line, recent efforts to identify ULK1 substrates in an unbiased manner have somewhat clarified the scope of ULK1 signalling and also opened new lines of study [74]. Egan *et al.* aimed to define the consensus phosphorylation motif that is recognized by ULK1 by screening a peptide substrate library *in vitro* with active purified ULK1 complex. This approach led to the formulation of an optimal ULKtide sequence (YANWLAA**S**IYLDGKKK) that features, for example, preferences for hydrophobic residues Met or Leu at the −3 position and aromatic residues like Tyr at the +2 position. Properties of the ULKtide bear some resemblance, for example, at the −3 and +2 positions, with the consensus motif described for yeast ATG1 [128]. Although the 10-residue-long ULKtide sequence still displays considerable variation, it provides a starting point to search genomic databases and also within given candidate proteins. Experimentally, robustness of the ULKtide consensus could be confirmed through cross-comparison with phospho-mass spectrometry data generated following co-expression of ULK1 with candidate substrates. In doing so, the authors were able to identify two new sites in ATG101 (along with multiple sites in ATG13 and FIP200) phosphorylated by ULK1. Similar approaches identified phosphorylation events on Beclin 1 (including S15, human), Ambra1 and VPS34. Generally these ULK1-directed phosphorylation events occurred on sites resembling the ULKtide, for example, with hydrophobic residues at −3. These results underscore how a network of phosphorylation events emanate downstream of ULK1, even within the subunits of the ULK and Beclin complexes. While these phosphorylation events have been identified (and characterised to a certain degree by mutagenesis), functional roles for the majority remain unclear, highlighting the challenge in defining specific events when multiple signals likely cooperate together. Nonetheless, definition of phosphorylation recognition patterns and specific inhibitors, coupled with existing genetic approaches, will allow for more thorough investigations of the full range of ATG1/ULK1 function.

## 4. Amino Acid Signalling and MTORC1-Dependent Autophagy

**Figure 3:**
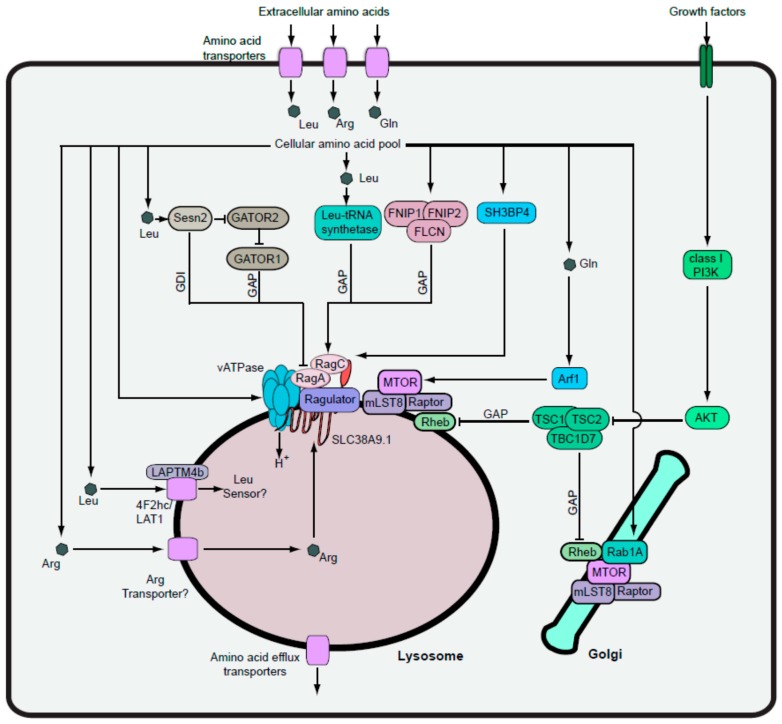
**Circuitry of amino acid signalling to MTORC1**. Extracellular amino acids are first transported into the cell. Regulatory amino acids such as Leu and Arg are further transported into the lysosome. Lysosomal Arg is sensed via SLC38A9.1, leading to activation of the vATPase-Ragulator complex and MTORC1. Leu is sensed via Sesn2 to regulate the GATOR1 pathway. Gln activates MTORC1 via an Arf1-mediated pathway. Amino acids also activate MTORC1 at the Golgi via Rab1A. Growth factor signalling leads to activation of PI3K and AKT, which controls the TSC1/2 complex and Rheb.

To better understand the full context, it is necessary to consider how autophagy regulation is integrated into cellular energy homeostasis. Our discussion has highlighted the overall primacy of amino acid-dependent MTORC1 signalling for the negative regulation of autophagy. Amino acid availability activates MTORC1, which stimulates protein translation and cell growth, while suppressing at the same time autophagy (see [144] for overall review). The area of MTORC1 regulation, in particular, has witnessed a tremendous expansion in molecular detail and inter-connectivity fuelled in part by unbiased proteomic searches. Below, we discuss major advances in MTOR signalling, focusing on the positive and negative regulatory networks that sense amino acid availability (summarised in Figure 3).

### 4.1. Uptake of Regulatory Amino Acids into Cells

In the basal state, resting mammalian cells encounter full, saturating levels of amino acids, glucose and growth factors (at least in the laboratory culture). Under these conditions, multiple pathways are engaged which converge, leading to MTORC1 activation. First, external amino acids enter the cell predominantly through the concerted function of members of the solute-linked carrier (SLC) family of transporters. Within the broad superfamily of membrane transporters, members have been historically categorised based on functional contexts, although HUGO more recently has provided the systematic SLC nomenclature. In the regulation of MTORC1 and autophagy, it had been recognised earlier that amino acids can greatly differ in their potency [101,145]. Recent work has provided mechanistic insight into the stimulatory effects of Leu, Gln and Arg, which are three of the most potent amino acids that activate MTORC1 via distinct sensing pathways.

The import of Leu into cells is primarily driven by the l-type transporter family (LAT1-4) [146]. LAT1 and LAT2 represent the SLC7 sub-family and function as a heterodimer with 4F2hc (4F2 antigen heavy chain). In contrast, LAT3 and LAT4 represent the SLC43 sub-family and function as low affinity transporters. Arg import is driven by cationic transporters, such as CAT-1, -2A, -2B and -3, which are all part of the SLC7 sub-family [147]. Also, Arg is transported via other routes such as the system y+L 4F2hc/y+LAT2 heterodimer [148]. On the other hand, Gln is transported through the concerted action of at least four different systems, some of which overlap with the transport of other amino acids. From these mechanisms, there is redundant, ubiquitous and thus robust maintenance of cytoplasmic Gln levels across wide cell contexts [149]. Transport of the regulatory amino acids can be inter-dependent. For example, sufficient cytoplasmic Gln levels are required by 4F2hc/LAT1 (SLC7A5) to drive uptake of Leu via bi-directional amino acid exchange [150]. As such, with Gln deprivation, cytoplasmic Leu subsequently becomes depleted leading to MTORC1 inactivation and autophagy, highlighting the complexity that needs to be recognised when attempting to pinpoint specific causal relationships within the metabolic network.

### 4.2. MTORC1 Activation via the Lysosomal Inside-Out Mechanism

Once cells achieve sufficient levels of cytoplasmic Leu, Gln and Arg, one of the major routes for activating MTORC1 occurs via an inside-out mechanism that involves coordinated input from multiple protein complexes in the lysosomal membrane [151]. MTORC1 itself contains a core module of MTOR, Raptor (regulator-associated protein of mTOR), and mLST8 (mammalian lethal with SEC13 protein 8). Understanding regarding the 3D organisation of this core complex has been particularly advanced via cryo-electron microscopy that illustrates how MTORC1 assembles with a symmetrical dimeric architecture and how specificity and access to the active site is controlled via interactions with Raptor [152,153]. The core MTORC1 is further regulated by additional non-core subunits, such as PRAS40 (proline-rich Akt substrate, 40 kDa). PRAS40 normally binds and inhibits MTORC1 but phosphorylation of PRAS40 by AKT outlines an additional mechanism linking growth factors to MTORC1 activation [154]. Tti1 and Tel2 are other accessory subunits that can regulate the protein stability of MTORC1 [155].

In the current consensus model, MTORC1 activation is associated with relocation of the complex onto the cytoplasmic surface of the lysosome through interaction with Rag family GTPases [156,157]. These Rag proteins function as heterodimeric complexes that in their active state, contain GTP-bound Rag A (or B) associated with GDP-bound Rag C (or D), which then binds the Raptor subunit of MTORC1. Once at the lysosome, MTORC1 receives further signals by interacting with another stimulatory GTPase, Rheb (Ras homolog enriched in brain). One critical aspect is that Rag heterodimers are normally kept localised on the lysosome by binding the lysosomal-resident Ragulator complex consisting of: p18 (LAMTOR1/C11orf59); p14 (LAMTOR2/ROBLD3); MP1 (LAMTOR3/MAPKSP1); LAMTOR4 (C7orf59); and LAMTOR5 (HBXIP) [158]. Together, the Rag and Ragulator complexes, along with vacuolar-ATPase, coordinate to form an amino acid-sensitive docking site responsible for anchoring MTORC1 onto the lysosome [151]. This involvement of v-ATPase in MTORC1 signalling is intriguing in light of its more widely appreciated role as the proton pump maintaining lysosomal acidification. The precise mechanism remains uncertain but v-ATPase forms multiple interactions with Ragulator subunits. Under high amino acid availability, binding between the Ragulator and the v-ATPase V1 domain is disrupted, which speculatively might free the Ragulator to regulate Rags. Thus, v-ATPase provides one mechanism that is able to sense high amino acid levels leading to GTP-loading on RagA/B via GEF activity from the Ragulator [159]. Consistent with this model, the robust role of nucleotide binding on RagA/B has been demonstrated: knock-in mice that express constitutively GTP-bound RagA display nutrient-insensitive MTORC1 activation and are unable to activate a normal autophagy survival response following post-natal fasting [77].

The pathway involving Rags and Ragulator was termed inside-out since amino acid levels were being sensed within the lumen to activate MTORC1 on the cytoplasmic face of the lysosome [151]. In recent years, dissection of the components that sense intra-lysosomal amino acids have further uncovered a novel role for SLC38A9, a member of the amino acid transporter superfamily. Two independent studies were able to identify this connection using proteomic searches for factors co-precipitating with Ragulator components and Rag proteins [79,160]. A third group focused their attention on SLC38A9 based on its prior association to lysosomal fractions from proteomic studies [78]. Using SLC38A9 as bait, a proteomic search for interacting proteins thereby identified all five members of the Ragulator complex as well as the four Rag proteins. Critically, interaction with Rags and the Ragulator were specific to the SLC38A9.1 isoform, which contains a cytosolic-facing conserved N-terminal 110 amino acid region [79]. Other members, such as SLC38A9.2 and SLC38A9.4, which lack this N-terminal sequence, did not bind Ragulator subunits. In agreement, mass spectrometry analyses with other related transporter members such as SLC36A1, SLC38A1, SLC38A2 or SLC38A7 failed to pick up any Ragulator or Rag proteins [78,79]. Thus, interactions with the Ragulator pathway were specific for SLC38A9.1.

Functionally, multiple lines of evidence illustrate SLC38A9.1 to be a robust stimulator of Ragulator-dependent MTORC1 signalling. For example, knockdown of SLC38A9.1 suppressed MTORC1 activation by amino acids. On the other hand, forced expression of SLC38A9.1 sustained MTORC1 activity, even following amino acid starvation. Interestingly, the stimulatory effects from SLC38A9.1 overexpression were fully blocked by the dominan negative RagB/C heterodimer, indicating that SLC38A9.1 acted upstream of the Rag complex [79]. In contrast, inhibition of v-ATPase only partially blocked effects from SLC38A9.1 overexpression. Thus, SLC38A9.1 and v-ATPase might represent independent or parallel pathways that converge on the Ragulator.

For mechanistic details, SLC38A9.1 bound more strongly to mutant forms of RagB that were constitutively GDP-associated [79]. Also, amino acid stimulation tended to loosen interactions between SLC38A9.1 and the Ragulator complex. These data help construct a model in which high amino acid availability frees the Ragulator to act as a GEF for RagA/B. Intriguingly, further studies of SLC38A9.1 binding were able to delineate more precise roles for the regulatory amino acids Arg and Leu. Addition of either of these amino acids disrupted SLC38A9.1–Ragulator binding. However, CRISPR-mediated deletion of SLC38A9.1 only blocked MTORC1 activation in response to Arg stimulation. Surprisingly, cells lacking SLC38A9.1 were still responsive to Leu. Thus, evidence supports SLC38A9.1 to be preferentially an Arg sensor for MTORC1.

### 4.3. Transport of Amino Acids into the Lysosome

For the inside-out pathway, levels of regulatory amino acids were sensed from within the lysosomal lumen. As summarised above, basic amino acids such as Arg are imported into the cell via cationic family transporters. What are the mechanisms that further transport cytosolic amino acids into the lysosome and, secondly, do these play any regulatory roles? The SLC38A9.1 was shown *in vitro* using liposomes to indeed be capable of transporting Arg, Gln, (and also Asn) into the lumen, which suggests a potential role in equilibrating cytosolic and lysosomal amino acids [78,79]. However, rates and affinity of SLC38A9 were only moderate, suggesting that this pathway might not be able to fully account for transport in cells. These data suggest that SLC38A9 may function primarily as the Arg sensor/receptor rather than a high efficiency transporter, and so the route of Arg entry into the lysosome remains unclear.

As one conundrum, interactions between SLC38A9.1 and the Ragulator were responsive to Leu [79]. However, since Leu still activated MTORC1 in cells lacking SLC38A9.1, other sensor pathways existed. Leu import into cells is driven by the LAT1 and LAT2 transporters, and other recent data have been able to outline further components of the Leu-sensing pathway. In this mechanism, transport of cytosolic Leu into the lysosome was mediated by 4F2hc/LAT1, which is recruited into the lysosomal membrane through the action of lysosome-associated transmembrane protein 4b (LAPTM4b) [161]. Knockdown of LAPTM4b decreased transport of Leu into lysosomes, which resulted in decreased activation of MTORC1. Thus, mechanisms that ensure import of regulatory amino acids, such as Leu, into the lysosomal lumen are required for MTORC1 activation. In agreement with this, overexpression of PAT1 (also known as lysosomal amino acid transporter (LYAAT-1) or SLC36A1) promoted efflux of amino acids out of the lysosome lumen and suppressed MTORC1 [151]. Overall, lysosomal amino acid transport has better characterised in term of export into the cytoplasm following proteolytic digestion, for example, in the context of lysosomal storage disorders [162,163]. The intra-lysosomal Leu sensor has yet to be defined. However, we now better appreciate that MTORC1 activation is dependent on the balance of export and import of regulatory amino acids such as Leu and Arg into the interior of the lysosome where they interact with their respective sensor proteins.

In the larger context, we need to integrate the other amino acid-sensing mechanisms to coordinate with the internal lysosomal pathways. For example, leucyl-tRNA synthetase has been shown to act as a Leu-specific sensor by acting as a GAP to generate the active GDP-bound form of RagD [164] (presumably on the cytosolic surface of the lysosome). High amino acid availability also recruits the folliculin (FLCN)-FNIP1/2 complex to the lysosome where it acts as a GAP for RagC/D [165]. The FLCN-FNIP complex may have additional roles for the regulation of MTORC1 localisation at the lysosome [166]. SH3-binding protein 4 (SH3BP4) can also bind and regulate the Rag complex in response to amino acid availability [167]. The GATOR complex discussed below contains another Leu sensor for the Rag pathway. Together with the inside-out mechanism, these findings illustrate how the Rag complex integrates a wide range of amino acid-dependent signals towards MTORC1.

### 4.4. Rag-Independent MTORC1 Pathways

While Rag complexes form a central route towards MTORC1, several further studies have outlined the wider range of amino acid-signalling pathways. One set of work from Jewell *et al.* investigated MEFs lacking both Rag A and Rag B. In this way, Rag A/B double knockout blocked the ability of Leu and Arg to activate MTORC1, but unexpectedly, Gln was still able to trigger signalling. CRISPR/Cas9-mediated double inactivation of Rag A and B in HEK293 cells also produced the same selective inhibition of just Leu and Arg signalling. Gln still induced lysosomal translocation of MTORC1 in Rag A/B KO MEFs, but this was blocked by Bafilomycin A or concanamycin A. Thus, Gln signalling to MTORC1 still required v-ATPase and the lysosomal docking aspects of the mechanism. Further investigation of this Gln-signalling pathway were based on earlier observations from the same research group implicating the Golgi-regulatory GTPase protein, Arf1, in MTORC1 signalling. Inhibition of Arf1 by knockdown or Brefeldin A treatment blocked the glutamine signalling in RagA/B-deficient cells. In contrast, other methods that disrupted general Golgi trafficking (such as Golgicide A) did not block amino acid signalling, which suggested that Arf1 had a Golgi-independent function to sense Gln and direct MTORC1 to the lysosome.

Complementary to the Arf1 mechanism, an independent report highlighted an alternative pathway involving the Rab1 GTPase during amino acid signalling [168]. Similarly, this work initiated from analyses of Rag-independent contexts, in this case, using yeast deficient in either of the Gtr1 or Gtr2 Rag homologues. Using this system, a screen based on sensitivity to rapamycin was able to identify Ypt1 (yeast homologue of Rab1A) in the TOR pathway. Interestingly, Ypt1 was required for amino acid-dependent activation of TORC1. Furthermore, Ypt1 co-precipitated with yeast TOR1 in an amino acid-dependent manner and amino acid stimulation promoted GTP loading of Ypt1. Based on these data, knockdown approaches using HEK cells demonstrated that mammalian Rab1A was essential for MTORC1 activation by amino acids. A role for Rab1A transmitting amino acid signals is intriguing given its better known role in coordinating membrane traffic from the ER [169]. However, the amino acid–Rab1A mechanism seems highly conserved. Similar to yeast, amino acid stimulation of mammalian cells promoted GTP-loading of Rab1A– and Rab1A–MTORC1 interactions.

### 4.5. Coordination of MTORC1-Activation Pathways

How does the Rab1A mechanism fit in with the other established MTORC1 pathways? As one clue, Rab1A knockdown inhibited MTORC1 activation driven by overexpression of Rheb. Conversely, knockdown of Rheb blocked the ability of Rab1A to stimulate MTORC1, thus showing mutual co-dependencies. Mutant versions of Rab1A that were either locked in the GDP-bound inactive state or lacking membrane localisation signals failed to activate MTORC1. A proximity ligation assay further demonstrated that Rab1A–MTORC1 interaction preferentially occurred at Golgi membranes. This approach also highlighted Rheb and MTORC1 interactions at the Golgi. Lastly, Rab1A knockdown specifically disrupted the Rheb–MTORC1 interaction (but had no effect on MTORC1-binding RagC). All these data suggest another layer to the MTORC1 signalling model in which Rab1A drives recruitment of MTORC1 and Rheb to form an activated complex on the Golgi. From this, we can conclude that at least two highly conserved mechanisms can promote MTORC1 activation but at distinct membrane sites, namely the lysosome and Golgi.

The relative contributions of lysosomal *vs.* Golgi MTORC1 pathways are still unclear. However, both these mechanism include key roles for GTP-bound Rheb during MTORC1 activation. There is indeed evidence supporting Rheb at various membrane locations, including the Golgi and lysosomes, but this issue is also controversial [156,170,171]. The Arf1 mechanism is interesting as it seems to be preferentially linked to the lysosomal MTORC1 pathway. Further below, we discuss the TSC pathway which also focuses on Rheb and MTORC1 at the lysosome. In the wider context, both the Arf1 and Rab1A mechanisms were discovered through searches of Rag-deficient cellular systems, thus outlining three pathways controlled by small GTP-binding proteins that work independently. However, Rab1A knockdown also had inhibitory effects on RagB/C-driven MTORC1 signalling [168], indicating some crosstalk not yet fully understood. It also remains unclear how the Rab1A–MTORC1 pathway might sense different amino acids. Rab1A has been shown to bind ATG1/ULK1 in both yeast and mammalian systems and this may reflect another mechanism distinct from MTORC1 [172]. Lastly, Arf1 and Rab1A would be predicted to control autophagy initiation via MTORC1, but how this is coordinated with the trafficking roles of these two GTPases remains unclear.

### 4.6. MTORC1 Shutdown via the GATOR Complex

As nutrient availability decreases, MTORC1 signalling is suppressed, leading to autophagy. Part of this mechanism involves reduction of the positive regulatory signals from the Ragulator, Arf1 and Rab1A pathways. In addition, pathways that negatively regulate MTORC1 have been identified in recent years revealing the network of counterbalance systems that also can sense amino acid availability. For example, Rag proteins receive critical negative regulation from the GATOR (GTPase-activating protein activity towards Rags) super-complex, which is comprised of the GATOR1 and GATOR2 sub-complexes. GATOR1 (consisting of subunits DEPDC5, Nprl2 and Nprl3) functions as a GAP for Rags A/B to inhibit MTORC1 signalling [173]. The importance of DEPDC5 and Nprl2 were underpinned by cases of mutation in their respective genes from glioblastoma and ovarian cancers. Loss of heterozygosity observed with these further suggested that GATOR1 functioned as a tumour suppressor. Thus, in tumour cells without GATOR1 (in agreement with GATOR1 knockdown), RagA/B and MTORC1 signalling were hyperactive and resistant to shutdown, even in the absence of amino acids.

The GATOR2 complex (consisting of Mios, WDR24, WDR59, Seh1L and Sec13) brings an additional layer of regulation by binding GATOR1 [173]. Through this interaction, GATOR2 suppresses the GAP function of GATOR1 towards RagA/B, thereby positively regulating MTORC1. A series of further reports that combine biochemistry, cell biology and structural biology have outlined a mechanism in which GATOR2 controls RagA/B and MTORC1 activation in response to Leu levels sensed via Sestrin family members [174,175,176,177,178]. Sestrins (Sesn1–Sesn3) are a group of highly conserved proteins that are induced as part of the p53- and FoxO-dependent stress-response (see [179] for review). As one role, Sesn1/2 lower levels of ROS accumulation inside cells, although the mechanism for this remains controversial [177,180,181]. From earlier data, Sesn1/2 were also implicated in MTORC1 signalling by decreasing the GTP-loading ratio of its activator, Rheb [182]. In this pathway, Sesn1/2 promoted AMPK-mediated phosphorylation of the TSC2 (tuberous sclerosis 2) protein and activation of the TSC1–TSC2 GAP complex towards Rheb.

Later studies indicated further novel aspects to the mechanism as Sesn2 overexpression suppressed MTORC1 function even in AMPK-null cells. Searches for Sesn2-interacting proteins recovered GATOR2 components [174,176]. Conversely, an independent group searching for GATOR2-interacting protein identified Sesn2 (in addition to Sesn1 and Sesn3) [175]. Consistent with overexpression trends, when Sestrin expression was targeted, MTORC1 tended to show higher levels of activation, both under amino acid-depleted and replete conditions [174,175]. Thus, all evidence point to Sestrins as negative regulators or MTORC1 signalling. Fitting into the larger mechanism, Sesn2 overexpression required functional GATOR1 to inhibit MTORC1. In addition, Sesn2 overexpression was able to disrupt interactions between GATOR2 and GATOR1 complexes, and furthermore, promote formation of GDP-bound RagB [176]. These findings help define a model in which Sestrins bind GATOR2, thereby releasing GATOR1 and enabling GAP activity towards RagA/B (leading to shut-down of MTORC1).

How is this mechanism regulated by amino acids? As one indication, Sesn2–GATOR2 interaction was strengthened following starvation of amino acids [175]. Further work narrowed this down to show that add-back of Leu alone could robustly disrupt binding between GATOR2 and Sesn2 (or Sesn1) [178]. This effect could be demonstrated following addition of Leu either to cells or *in vitro* on purified Sens2–GATOR2 complexes. Other amino acids similar to Leu (such as methionine and isoleucine) could also disrupt Sesn2–GATOR2 binding but with much less potency. Importantly, biochemical approaches could show direct binding of Leu to purified Sesn2 and a crystal structure was solved for Sesn2 in complex with Leu [177], illustrating the network of interactions leading to specific recognition of Leu at a single binding pocket of Sesn2. Mutant versions of Sesn2 (with either L261A or E451A substitutions) were found to bind Leu poorly and, as predicted, these Sesn2 variants could no longer activate MTORC1 following stimulation with Leu. While Sesn2 may act as a Leu sensor upstream of GATOR2, additional pathways integrate into the larger scheme. A complementary mechanism has been suggested, as Sestrins also have the ability to directly bind Rag complexes and act as a guanine nucleotide dissociation inhibitor (GDI) for RagA/B to overall suppress MTORC1 activation [183]. For this function, a conserved motif could be identified in mammalian and *Drosophila* Sestrins that was similar to that of Rab GDI, and mutation of key charged residues within this motif abrogated Sestrin GDI activity and the ability to suppress the MTORC1 pathway. Altogether, a rapidly expanding body of evidence indicates that Sestrins may have separate AMPK-, GATOR- and GDI-dependent signalling mechanisms that integrate to suppress the MTORC1 pathway.

As Sestrins inactivate RagA/B and MTORC1 following amino acid starvation and other types of stress, they would be postulated to promote autophagy. Indeed, forced expression of Sesn2 stimulates autophagy in renal tubule cells [184]. Conversely, knockdown of Sesn2 in p53-containing cells such as HCT116 and U2OS suppressed starvation-induced autophagy [185]. Interestingly, Sesn2 knockdown also suppressed MTORC1-independent autophagy caused by lithium, which reinforces the multiple pathways linked to Sestrins. Another intriguing finding is that Sesn2 also binds the EAT region of ULK1 and this interaction can promote ULK1-directed phosphorylation of p62/SQSTM1 at a critical serine 403 regulatory site [124]. Speculatively, the Sestrin–ULK1 interaction may be promoting p62/SQSTM1-mediated autophagy of Keap1 as part of an oxidative stress response [186]. ULK1 also can phosphorylate Sesn2 on multiple sites along the protein, although the function of these events remains unclear. These data suggest that Sestrins have the potential to modulate autophagy via MTORC1-dependent and -independent routes, which may be linked to the wide range of Sestrin function-sensing amino acids and other forms of cellular stress.

### 4.7. MTORC1 Shutdown via the TSC Complex

A central component in the model for MTORC1 activation features interaction with active GTP-bound Rheb. In agreement, pathways that lead to Rheb inactivation also serve as negative regulators of MTORC1 signalling. The intrinsic GTPase activity of Rheb is promoted by the TSC complex, made up of TSC1, TSC2 and TBC1D7 (tre2-bub2-cdc16-1 domain family member 7) [187]. TSC2 contains the GAP activity for Rheb while TSC1 serves as a scaffold for TSC2 and TBC1D7, stabilising the protein complex. This mechanism is widely recognised as the basis for growth factor-mediated regulation of MTORC1. For example, insulin stimulation activates the PI3K/AKT pathway leading to phosphorylation of TSC2 on at least five regulatory sites thereby inhibiting its function [188,189]. More recently, it was shown that these phosphorylation events cause dissociation of the TSC1/2 complex from the lysosome, thereby displacing its activity away from Rheb [171]. As such, growth factors in effect protect Rheb from inactivation, thereby promoting MTORC1. Conversely, growth factor (or serum) deprivation allows Rheb inactivation, MTORC1 shutdown and autophagy.

While regulation of TSC1/2 by AKT and growth factors has been established, other findings indicate additional levels of inter-connectivity, with amino acid-dependent recruitment of the TSC complex to the lysosome. Proteomic approaches discovered that TSC2 precipitated with RagA + RagC heterodimers [190]. Interestingly, binding was strongest with inactive Rag conformations associated with amino acid starvation (*i.e.*, RagA-GDP and RagC-GTP). In agreement, amino acid starvation promoted translocation of the TSC1/2 complex to the lysosome, and functionally, this recruitment was critical to drive MTORC1 release. In TSC2 −/− MEFS, MTORC1 remained stuck on the lysosome and MTORC1 activation persisted even following amino acid starvation. How does TSC2 control MTORC1 release? Since TSC2 is a Rheb GAP, the authors tested this potential link and were able to demonstrate that the persistent MTORC1 localisation at the lysosome following TSC2 loss could be reversed by knockdown of Rheb. These results thus suggest a revised model in which both Rag and Rheb proteins contribute towards anchoring MTORC1 at the lysosome for activation. Critically, under starvation conditions, inactive Rag heterodimers, help recruit the TSC1/2 complex to then inactivate Rheb and allow MTORC1 dissociation. Further results in this study illustrate how the TSC complex is essential to mount a proper pro-survival autophagy response following extended periods of amino acid starvation. Requirements for the TSC1/2 complex to suppress MTORC1 and promote autophagy have been observed in a number of contexts [191,192]. With the more recent data, it is better understood how the TSC complex also senses amino acid signals via the Rag pathway to regulate the lysosomal Rheb-MTORC1 pathway upstream of autophagy.

## 5. Summary of MTORC1 Pathways and Autophagy

Recent years have witnessed substantial development in our understanding of the nutrient-dependent pathways that control MTORC1. This finer detailing of the cellular metabolic networks thus outlines the wide range of pathways that channel in signals to modulate the autophagy homeostatic mechanism. Major advancements have been made in the identification of specific proteins that sense levels of regulatory amino acids such as Leu and Arg. A prominent concept is the role of the lysosome as a focal point for MTORC1 activation with the refinement of an inside-out mechanism in which amino acids from the lysosome interior are detected. A key new player in this pathway is the SLC38A9 membrane transporter protein which senses Arg and then transmits signals via v-ATPase and the Ragulator complex to activate MTORC1 on the lysosome outer surface. Pivotal to the Ragulator pathway are the Rag proteins localised on the lysosome, but other MTORC1 pathways can function independently, such as the Arf1-dependent mechanism, which preferentially senses Gln levels, while a Rab1A-dependent pathway preferentially activates Golgi-localised MTORC1. While mechanisms were identified that activate MTORC1, equally intricate counter pathways that suppress MTORC1 signalling were mapped out, including the GATOR1 GAP complex that inactivates RagA/B. Dissection of the GATOR1 function thus led to characterisation of the GATOR2 complex and its direct link to the Sestrin proteins which comprise another Leu-specific sensing pathway. The other key MTORC1 suppressor is the TSC1/2 complex which acts as a GAP for Rheb and the recent evidence indicates deeper levels of crosstalk, with inactive Rag proteins recruiting TSC1/2 to further inactivate Rheb.

Overall, these advances in nutrient-directed signalling pathways provide a more comprehensive list of molecular players that may serve as drug targets or genetic polymorphism candidates, for example, in the context of autophagy and cancer. Given the wide array of pathways that integrate amino acid and growth factor signals to MTORC1, it would be somewhat surprising if the link to autophagy initiation is confined to a small set of phosphorylation events on ULK1/2. It was outside the scope here, but several MTORC1-dependent mechanisms have been described for the up-regulation of lysosomal capacity, including the TFEB gene expression programme [193,194,195]. It is also intriguing that MTORC1 regulation is centred on the lysosome while autophagy membrane traffic ultimately funnels into the lysosome. Through prolonged autophagic flux and the re-generation of nutritional building blocks, the lysosomal network has also been characterised to tubulate, re-model and re-form via the autophagic lysosome reformation (ALR) programme driven by MTORC1 [196]. Thus, the lysosome is well-placed to provide a feedback mechanism that coordinates late stages of autophagy degradation with regulation during the early stages. Golgi-localised MTORC1 might have a different flavour of regulation, at least in terms of autophagy. Also, complementary to the amino acid-sensing pathways of MTORC1, decreased cellular energetics and AMPK are also accepted activators of autophagy [65,80,135], although this area is more controversial [197,198,199]. However, novel mechanisms have been proposed that link AMPK to the Ragulator [200], and as discussed earlier, AMPK signals are integrated with MTORC1 at the ULK1/2 complex and also at the Beclin 1-VPS34 complex. As such, AMPK is a critical regulator of autophagy but its role may be more context-specific.

We have structured our summary here into three broad areas, but nonetheless, all the mechanisms discussed work seamlessly in mammalian cells to activate and then terminate the autophagy response. If we zoomed out, we would see just the salient features: how starvation of key amino acids is sensed by a network of mechanisms to result in lower MTORC1 activity and how decreased MTORC1 activity leads to modified phosphorylation on ULK1 that triggers a translocation to autophagosome assembly sites associated with the endoplasmic reticulum. Activated ULK1 would then phosphorylate downstream substrates including members of the Beclin1 complex to drive further membrane trafficking and autophagosome assembly. From our discussion, we also appreciate that by zooming in, we uncover immense mechanistic diversity and complexity, reflecting the wide range of physiologic roles for autophagy under different contexts in mammalian cells. Teleological beauty continues to be discovered in the expanding world of autophagy biology. Pragmatically, our improved understanding of the regulatory circuitry also facilitates the development of pharmaceutical and genetic strategies for targeting autophagy, which will undoubtedly need fine-tuning across different biomedical contexts.
